# Supplementary figures and images for: Chemical genomics informs antibiotic and essential gene function in Acinetobacter baumannii
Source: PLoS Genet. 2025 Mar 28;21(3):e1011642. doi: 10.1371/journal.pgen.1011642 (PMC11975115; doi:10.1371/journal.pgen.1011642)

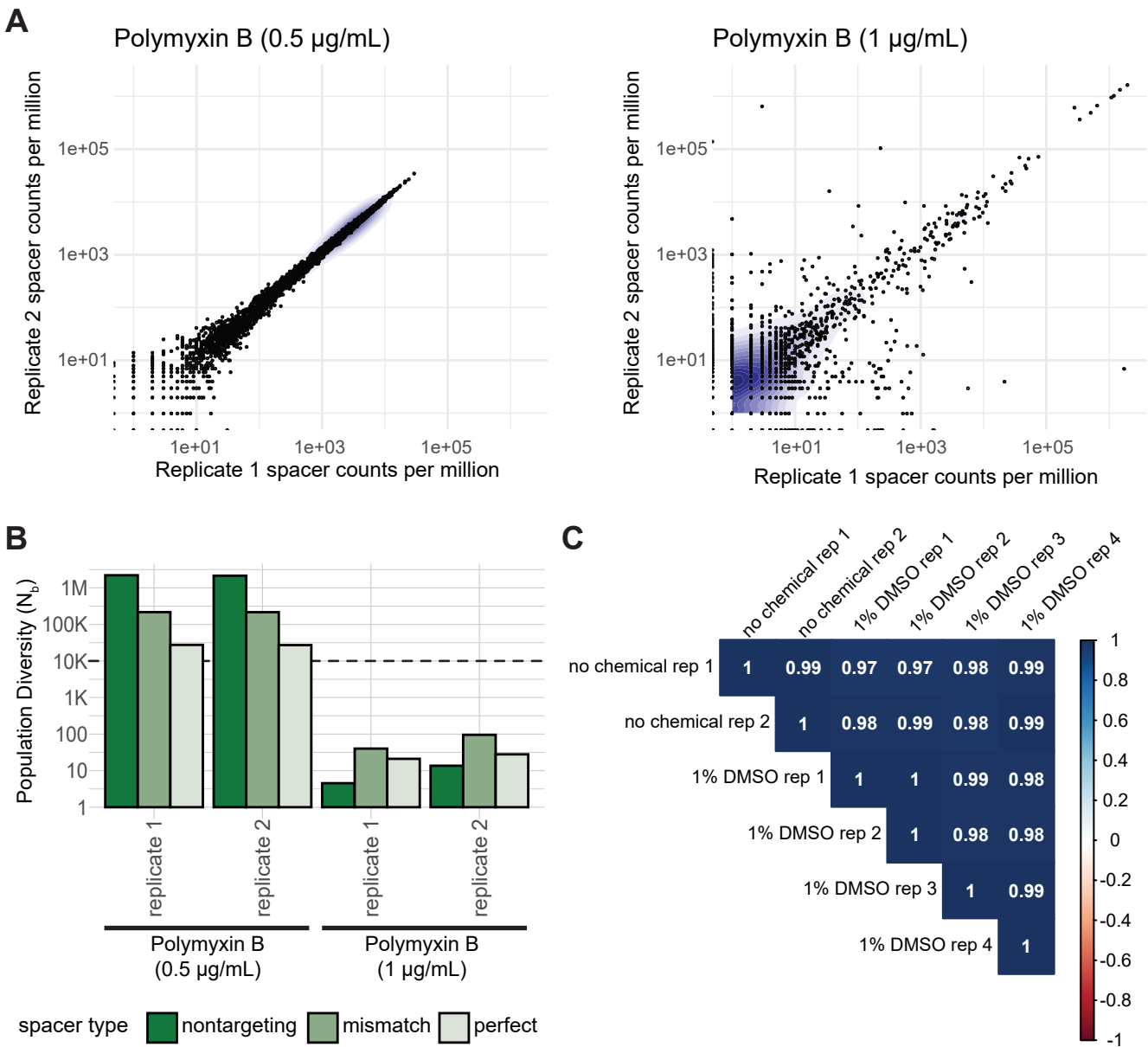

Supplement: S1 Fig — (A) Correlation of library spacer counts per million (CPM) across the two biological replicates for samples containing polymyxin B at concentrations of 0.5 ug/mL (left) or 1 ug/mL (right). Count density represented by contour map; right graph shows over-depletion of guides. (B) Population diversity (Nb) for polymyxin B replicates. Samples with nontargeting guide complexity below the cutoff (dotted line) were excluded from analyses. (C) Heatmap showing correlations of spacer CPMs between solvent controls. All solvent control samples show r≥0.97. (PDF) [file pgen.1011642.s001.pdf]

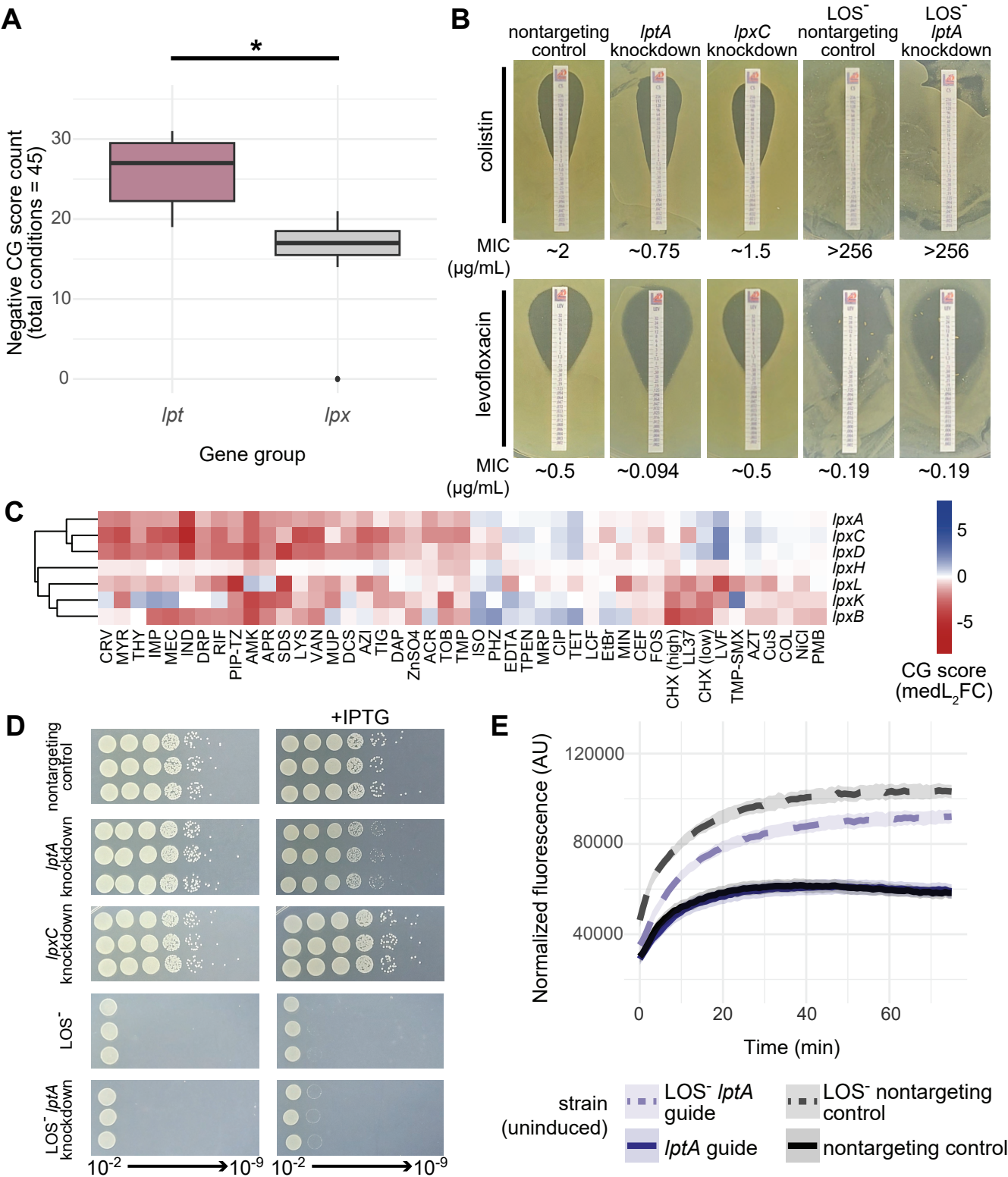

Supplement: S2 Fig — (A) Box plot of significant negative CG scores for lpt or lpx genes. Asterisk denotes p < 0.05 (Student’s t-test). (B) Colistin and levofloxacin MIC test strip assays for lptA or lpxC knockdown, LOS-, or nontargeting guide control strains. Plates supplemented with 1mM IPTG. Approximate MICs indicated below images. (C) Heatmap of CG scores of lpx knockdowns across conditions, showing muted phenotypes compared to lpt knockdowns. (D) 10 uL spots of ten-fold serial dilutions on plates with and without induction (N=3 biological replicates). The lptA knockdown, but not the lpxC knockdown, exhibits a minor growth defect upon induction. LOS- strains show major growth defects. (E) Ethidium bromide permeability assay for lptA knockdown and nontargeting control in 19606 or LOS- backgrounds without induction; increased fluorescence over time indicates membrane permeability. Ribbons represent standard deviation (N=4 biological replicates). Without induction, the strain containing the lptA guide behaves similarly to a nontargeting guide control. (PDF) [file pgen.1011642.s002.pdf]

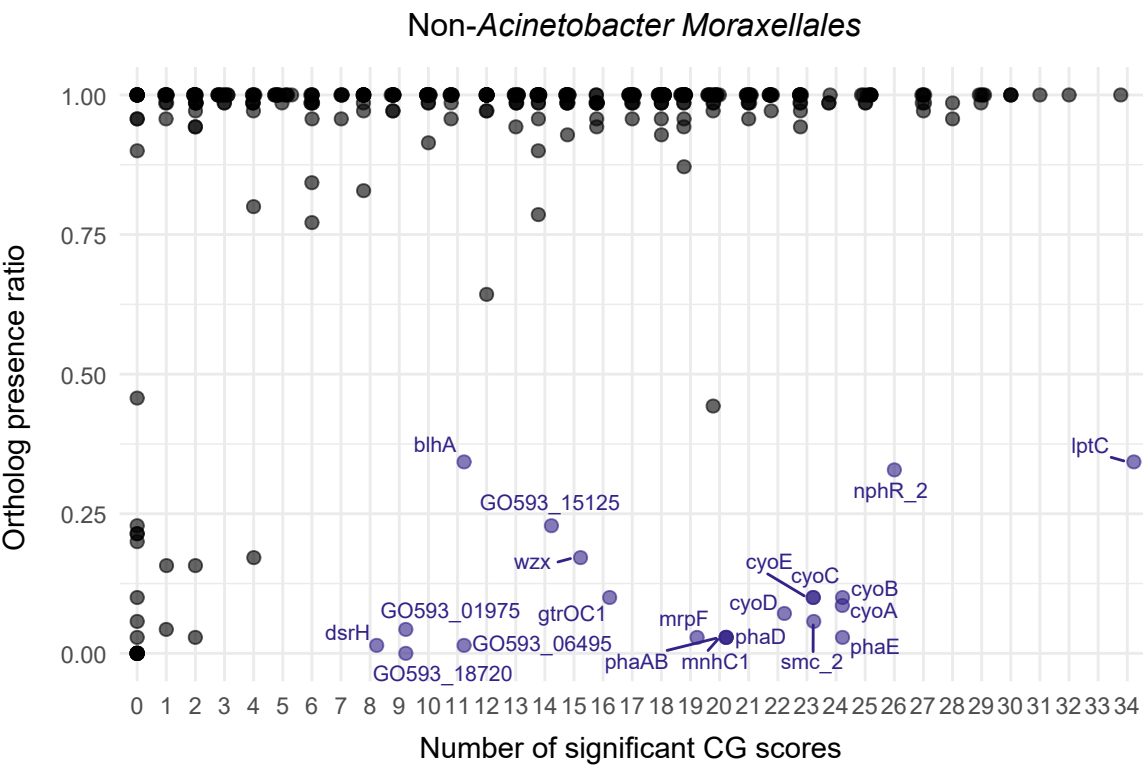

Supplement: S3 Fig — Dot plot depicting significant CG scores and ortholog presence ratio (fraction of isolates possessing at least one ortholog out of the total number of analyzed isolates) across representative non-Acinetobacter Moraxellales for library essential genes. Dots in blue represent genes with <38% presence across representative groups and significant chemical-gene interactions in >10% of screen conditions. (PDF) [file pgen.1011642.s003.pdf]

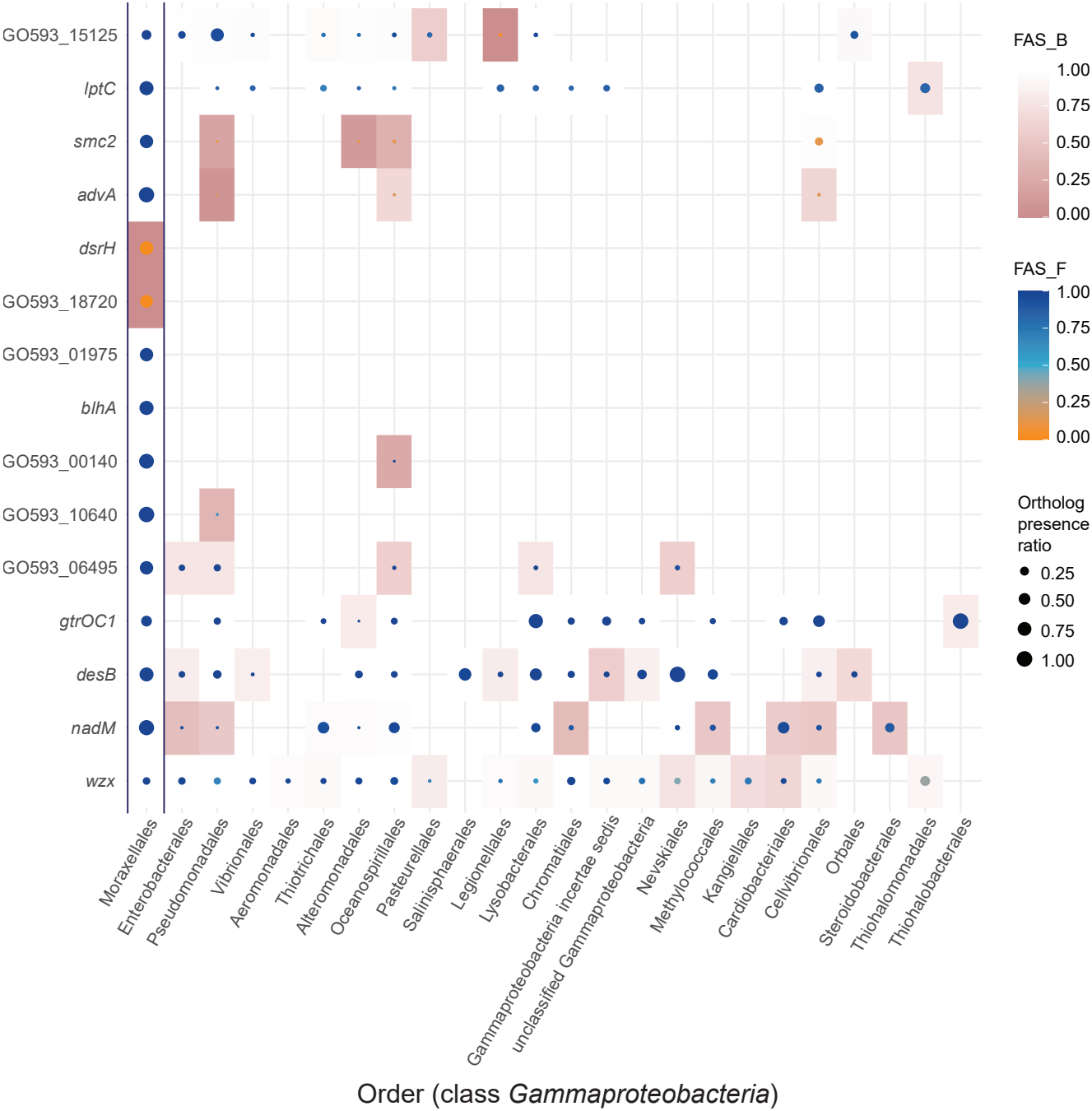

Supplement: S4 Fig — Phylogenetic profile of candidate genes showing ortholog presence ratios (fraction of isolates possessing at least one ortholog out of the total number of analyzed isolates; circle sizes) for selected A. baumannii genes across Gammaproteobacteria orders. The color encodes the median feature architecture similarity (FAS score) between the protein in 19606 and its orthologs within an order. The dot color gradient (FAS_F; blue to orange) captures architecture differences using the 19606 protein as reference. The cell color gradient (FAS_B; white to pink) captures architecture differences using the ortholog as reference. The score decreases if features in reference are missing in the respective orthologs. (PDF) [file pgen.1011642.s004.pdf]

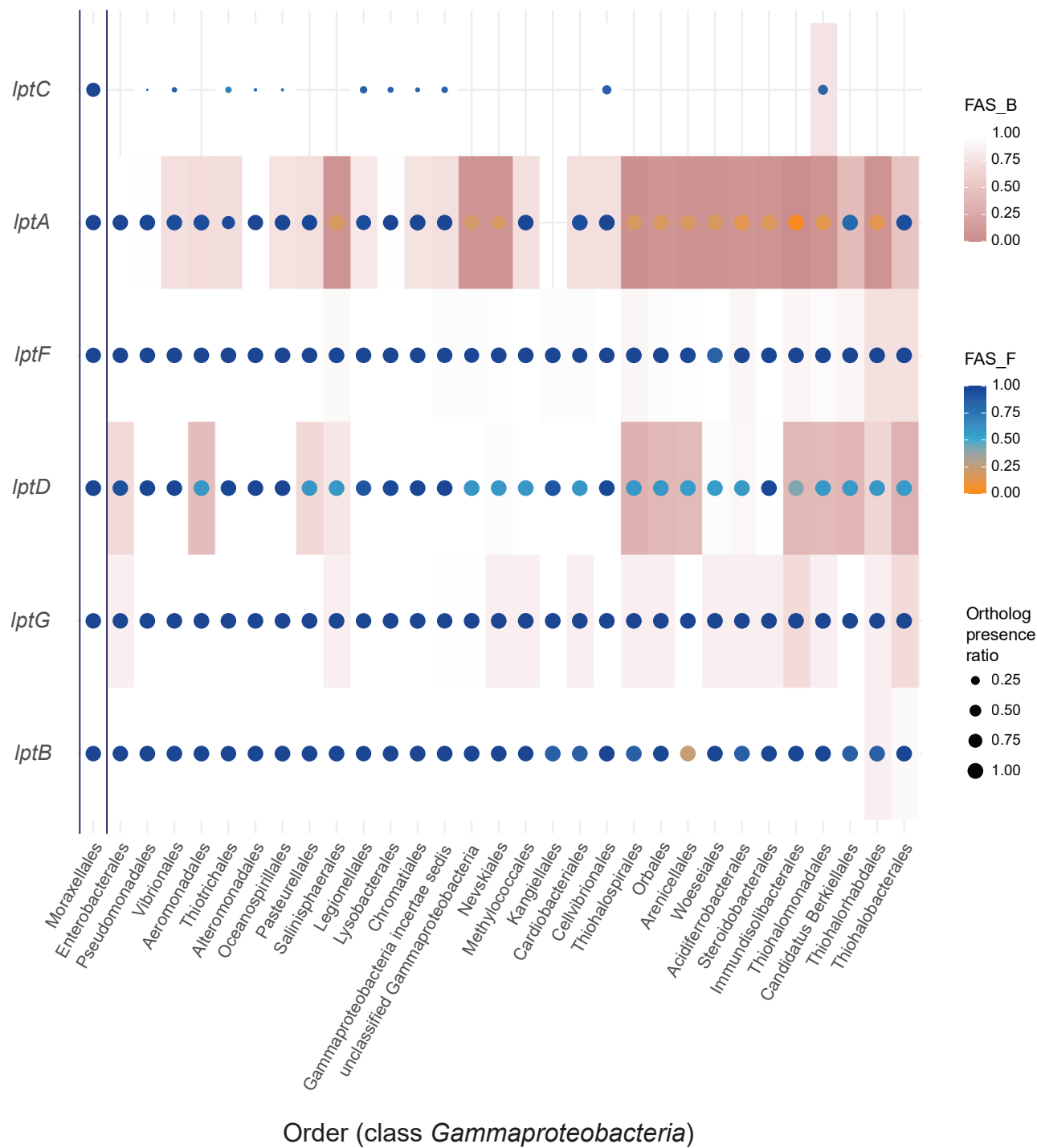

Supplement: S5 Fig — Phylogenetic profile of candidate genes showing ortholog presence ratios (fraction of isolates possessing at least one ortholog out of the total number of analyzed isolates; circle sizes) for selected lpt genes across Gammaproteobacteria orders. The color encodes the median feature architecture similarity (FAS score) between the protein in 19606 and its orthologs within an order. The dot color gradient (FAS_F; blue to orange) captures architecture differences using the 19606 protein as reference. The cell color gradient (FAS_B; white to pink) captures architecture differences using the ortholog as reference. The score decreases if features in reference are missing in the respective orthologs. (PDF) [file pgen.1011642.s005.pdf]

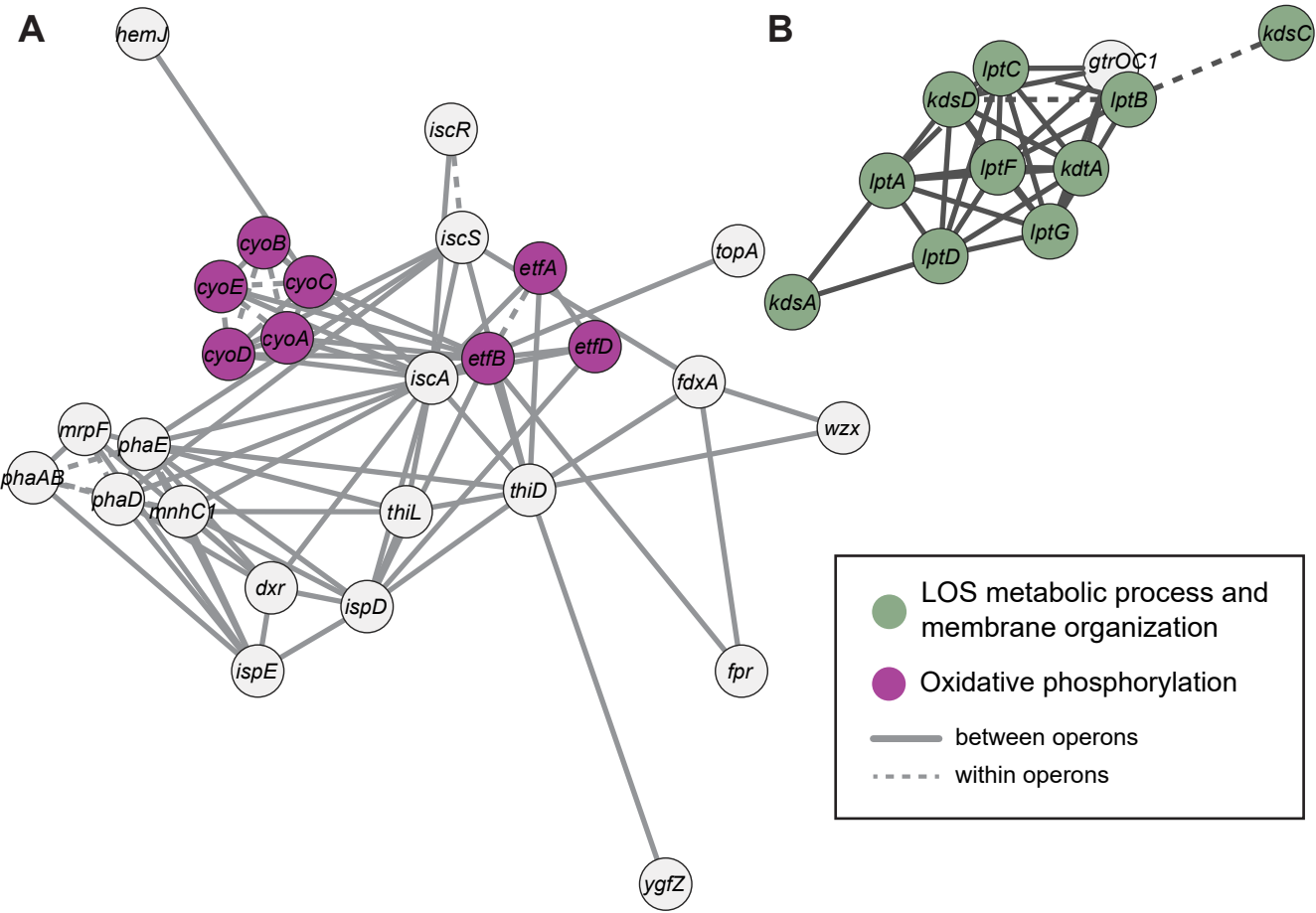

Supplement: S6 Fig — Sections from the essential gene network for (A) cytochrome bo3 oxidase (cyo), ion transporter (phaAB, mnhC1, phaD, mrpF), and related genes or (B) lpt-associated genes, including gtrOC1. Colors indicate STRING functional groups; solid or dotted lines indicate genes between or within operons, respectively. (PDF) [file pgen.1011642.s006.pdf]

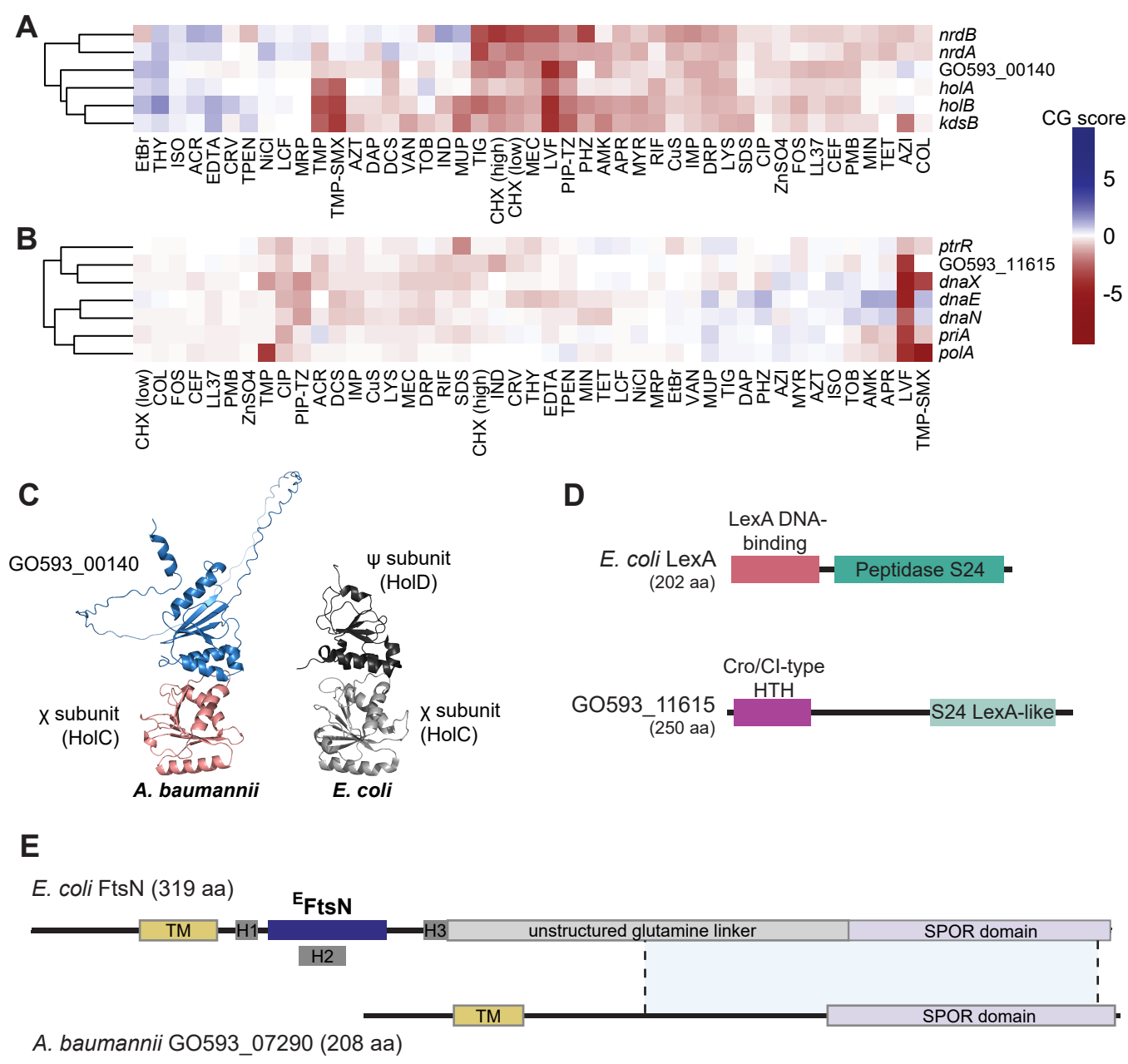

Supplement: S7 Fig — Heatmaps of CG scores for clusters containing (A) GO593_00140 or (B) GO593_11615. Y-axis clustering was conducted using the Ward method and Canberra distance across all library knockdowns. (C) Heterodimer structural prediction (Alphafold2) for GO593_00140 and the chi subunit of DNA Polymerase III in A. baumannii (left) and the solved structure for the E. coli psi-chi subunit heterodimer (right). GO593_00140 has a lengthy unstructured N-terminal end absent in E. coli HolD. (D) Domain architecture map from InterProScan analysis, comparing E. coli LexA to GO593_11615. Both have a DNA-binding domain and S24 peptidase/LexA domain but differ in overall protein length and specific type of DNA-binding domain. (E) Domain architectures for E. coli FtsN and A. baumannii GO593_07290. GO593_07290 lacks similarity to the short EFtsN region required for function (47). BLAST-aligned amino acid sequences (24% identity) are depicted with dotted lines. (PDF) [file pgen.1011642.s007.pdf]

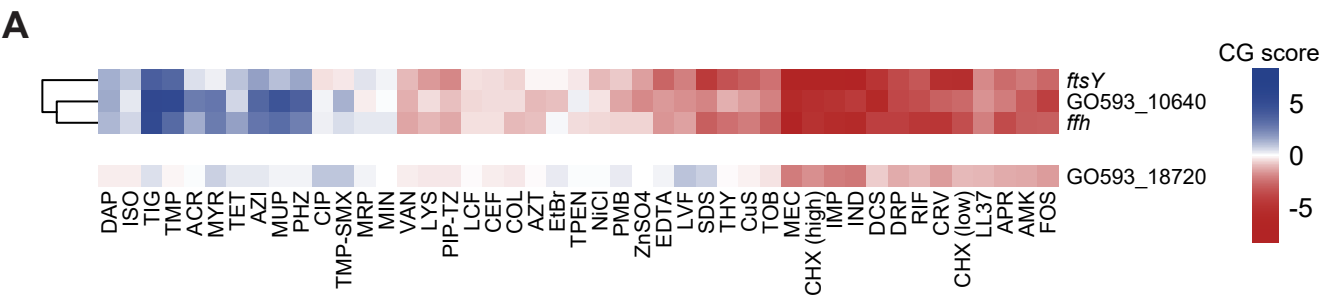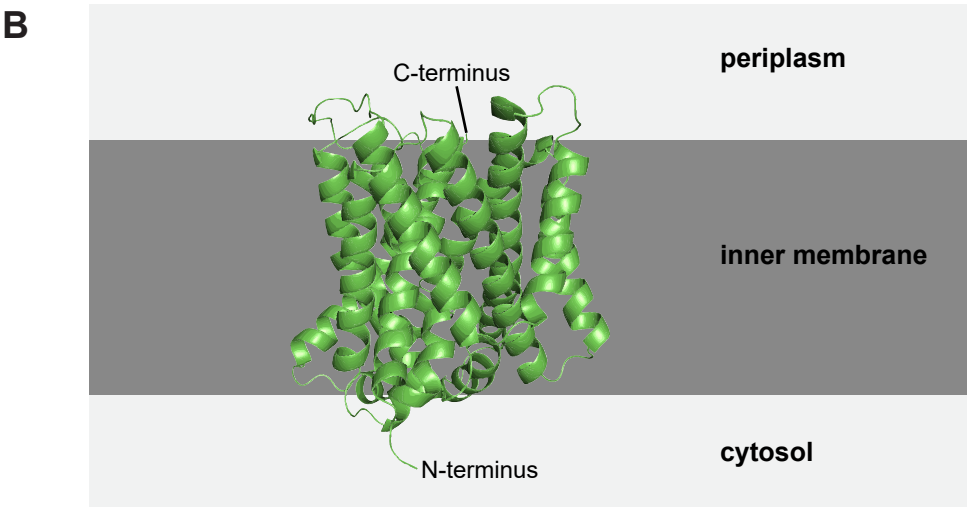

Supplement: S8 Fig — (A) Heatmaps display medL2FC for the signal-recognition cluster containing ftsY, ffh, and GO593_10640. GO593_18720 does not cluster in the heatmap with these genes due to muted phenotypes but is connected in the network. (B) GO593_10640 is a predicted transmembrane protein similar to a transporter. Predicted structure (Alphafold) and orientation in the membrane (TMHMM) are shown. (PDF) [file pgen.1011642.s008.pdf]

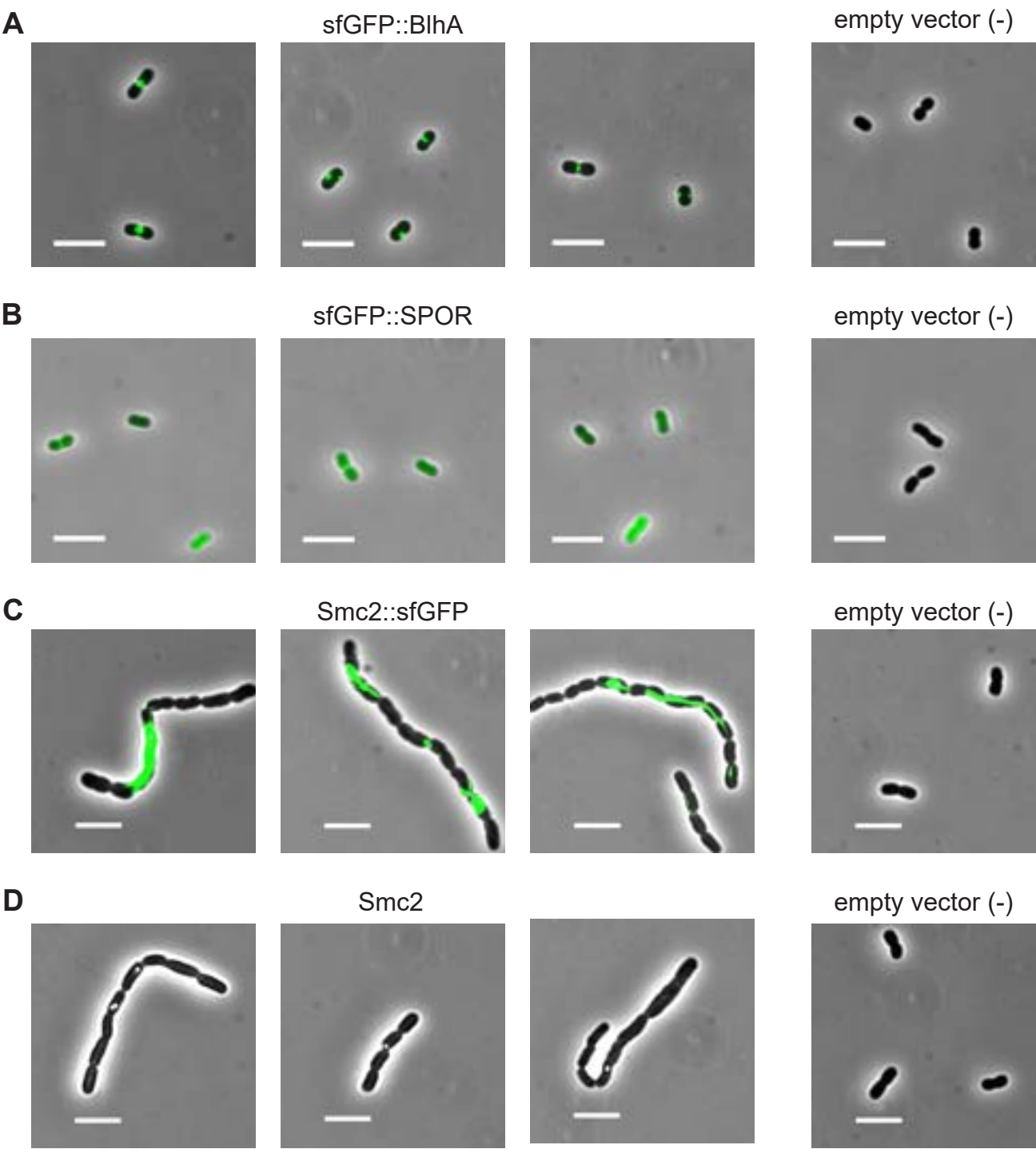

Supplement: S9 Fig — 3 representative images for each construct are shown with corresponding empty vector controls. (A) sfGFP::BlhA localizes at sites of division at the mid-cell at either existing or forming septa. (B) Fluorescently tagged SPOR-domain protein shows localization at the cell membrane and at septa for dividing cells. (C) Smc2::sfGFP expression leads to elongated cells, with fluorescently tagged proteins aggregating inconsistently within the cells. (D) Expression of Smc2 from a replicative plasmid produces elongated cells. All bars are 5 μm; GFP fluorescence/phase contrast channels merged in composite images for all except (D). (PDF) [file pgen.1011642.s009.pdf]

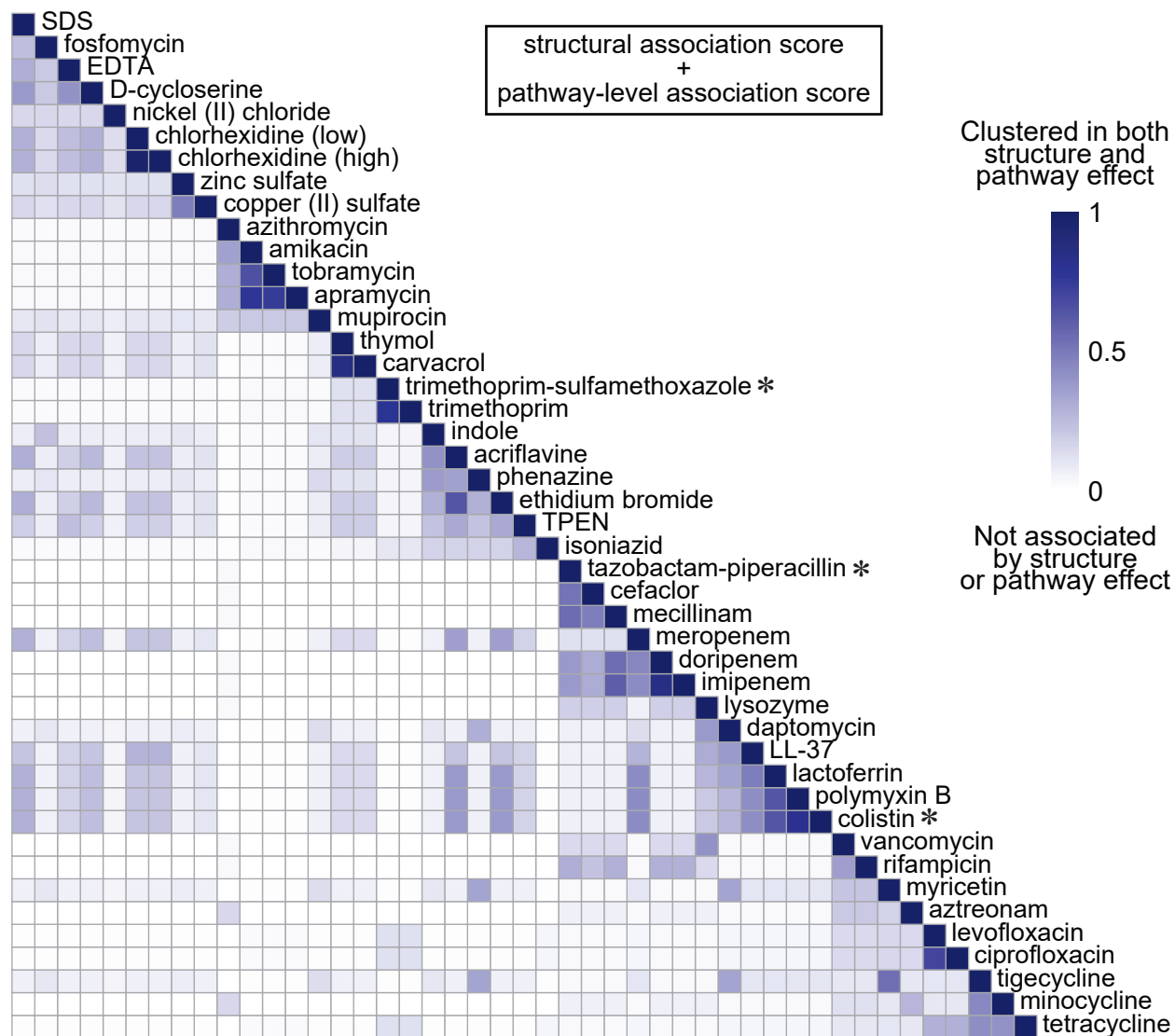

Supplement: S10 Fig — . Heatmap shows the normalized sum of pathway effect and structure association scores for chemical pairs. Axes are identical. Dark blue represents chemical pairs clustered tightly in both structure or pathway effect; white represents chemicals not clustered in either structure or pathway effect. Stars represent compounds or treatments with multiple chemical structures, where one was selected for structural comparisons (see S4 Table). (PDF) [file pgen.1011642.s010.pdf]

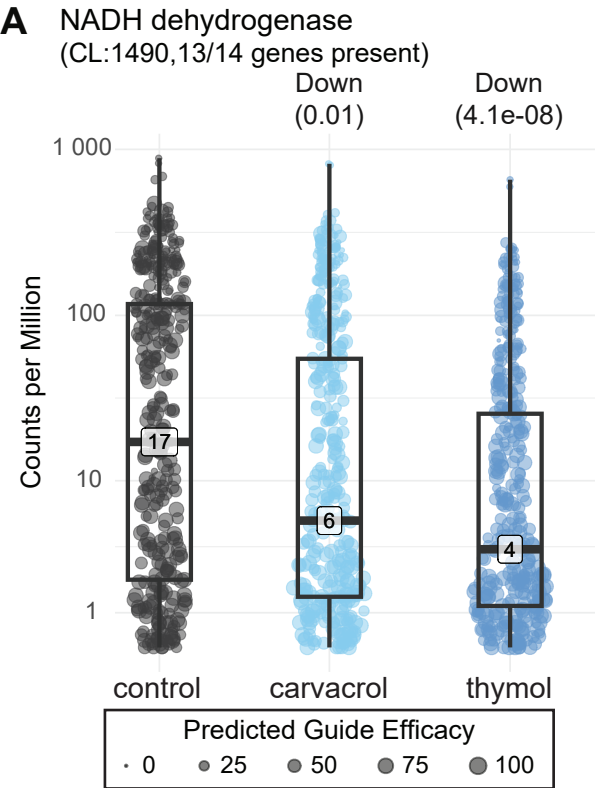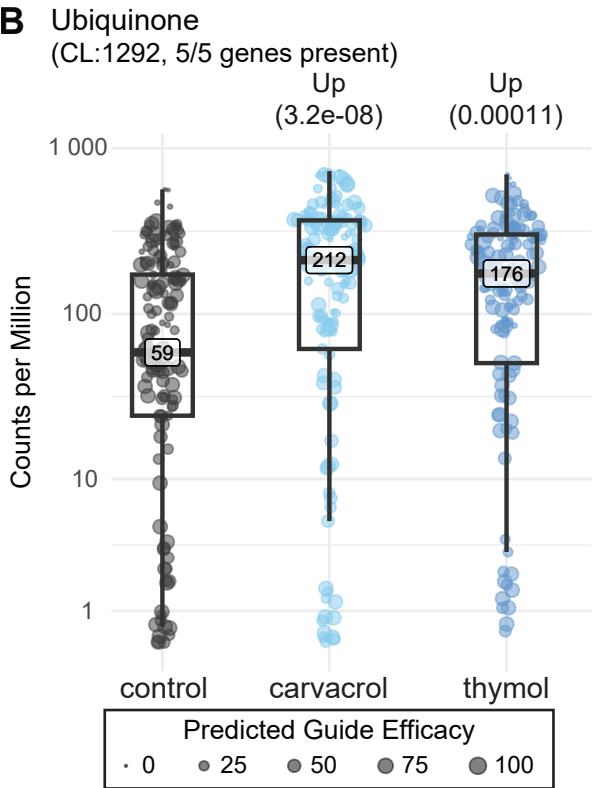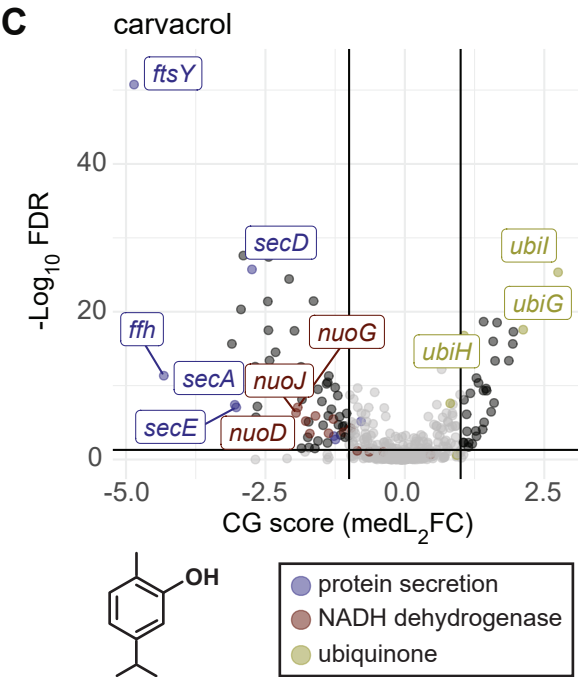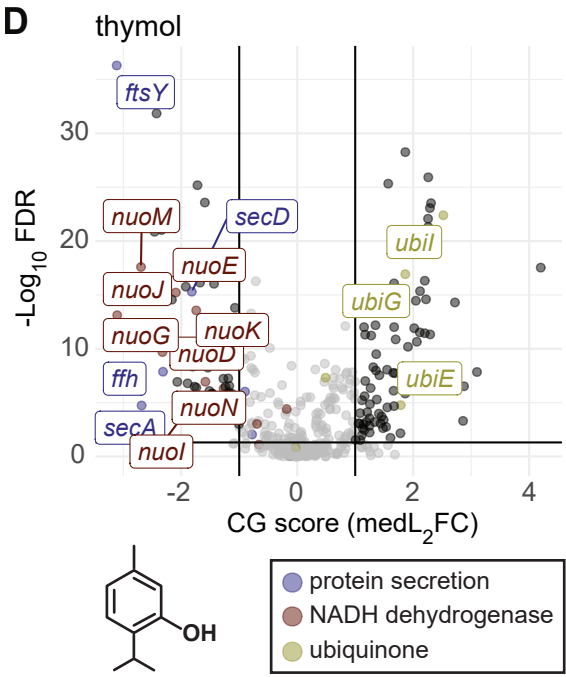

Supplement: S11 Fig — . Sina plots depict differential guide CPMs for (A) NADH dehydrogenase and (B) ubiquinone pathways in carvacrol and thymol. Chemical-gene interactions are described above each graph—up (positive), down (negative), or no change—with FDR values. Guides are weighted in this calculation by predicted efficacy, with perfect guides at 100. Volcano plots show relative fitness and FDRs at the gene-level in (C) carvacrol or (D) thymol. Genes in pathways of interest are highlighted, and chemical structures are depicted. (PDF) [file pgen.1011642.s011.pdf]

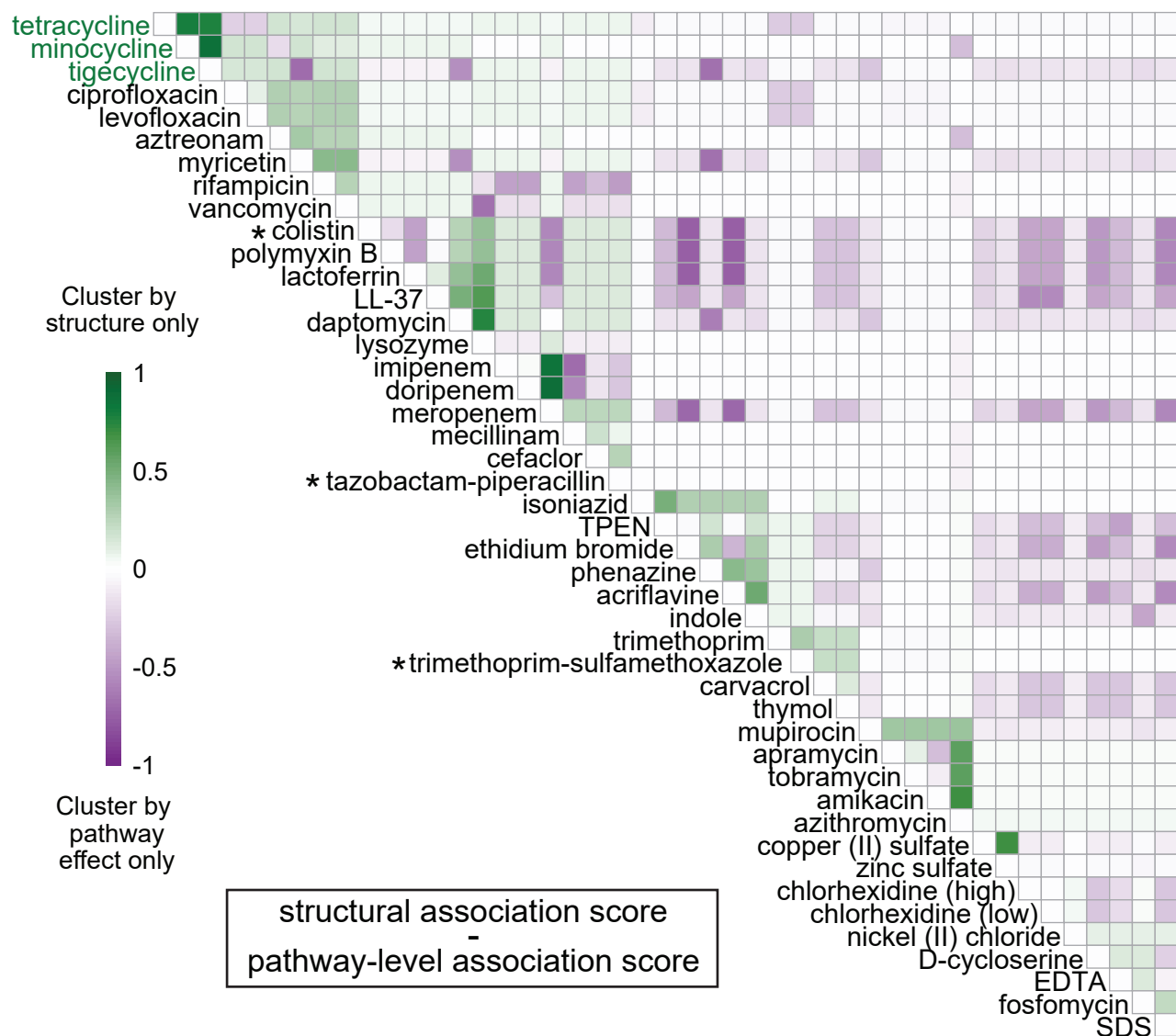

Supplement: S12 Fig — . Heatmap shows the normalized differences between pathway effect and structure association scores for chemical pairs. Axes are identical. Green represents chemical pairs clustered tightly by structure but not pathway effect; purple represents chemicals clustered by pathway effect but not by structure. Stars represent compounds or treatments with multiple chemical structures, where one was selected for structural comparisons (see S4 Table). (PDF) [file pgen.1011642.s012.pdf]

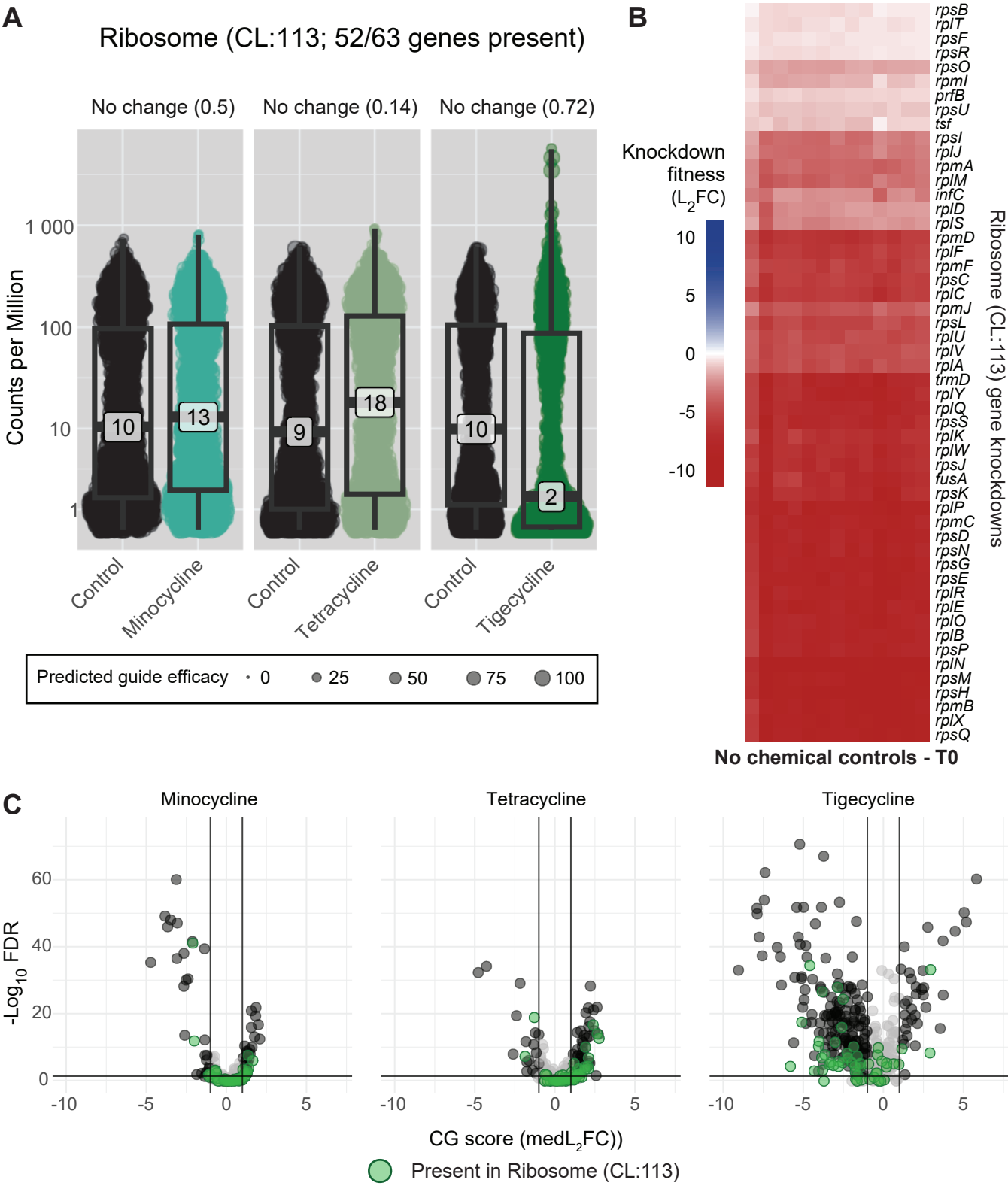

Supplement: S14 Fig — (A) Sina plots depict differential guide CPMs for ribosome genes (STRING identifier CL:113) in minocycline, tetracycline, or tigecycline compared to no chemical control. Chemical-gene interactions are described above each graph—up (positive), down (negative), or no change—with FDR values. Guides are weighted in this calculation by predicted efficacy, with perfect guides at 100. (B) Heatmap shows relative fitness scores(median log2 fold changes) of ribosomal gene knockdowns of the mock treatment control compared to T0. Ribosome knockdowns all have substantial loss of fitness without additional chemical treatment. (C) Volcano plots of library gene CG scores (median log2 fold change compared to mock treatment) in minocycline, tetracycline, and tigecycline. Genes in the ribosome group are colored green. Lines depict cutoffs for significance (|median log2 fold change| ≥1, Stouffer’s p < 0.05). (PDF) [file pgen.1011642.s014.pdf]

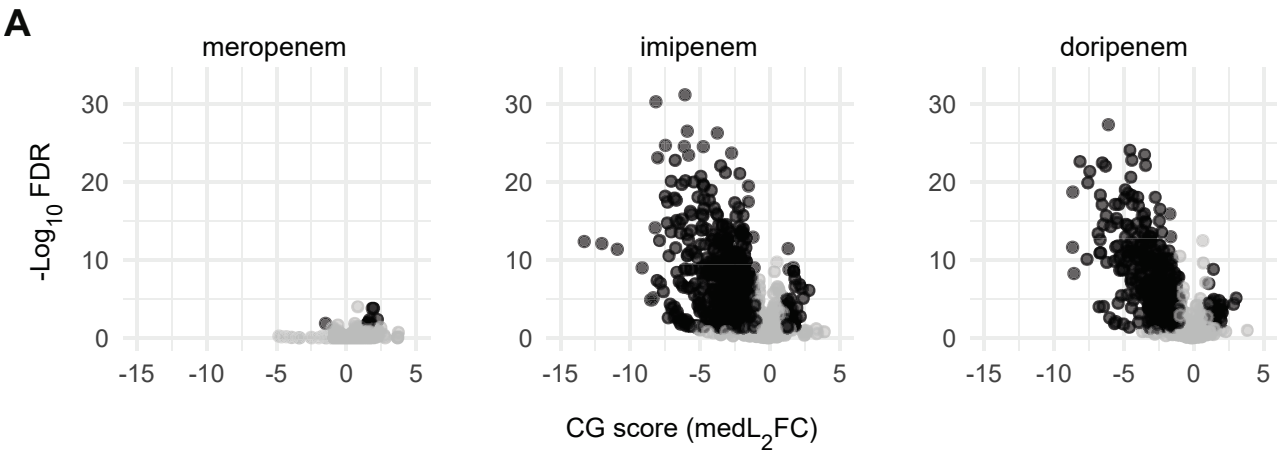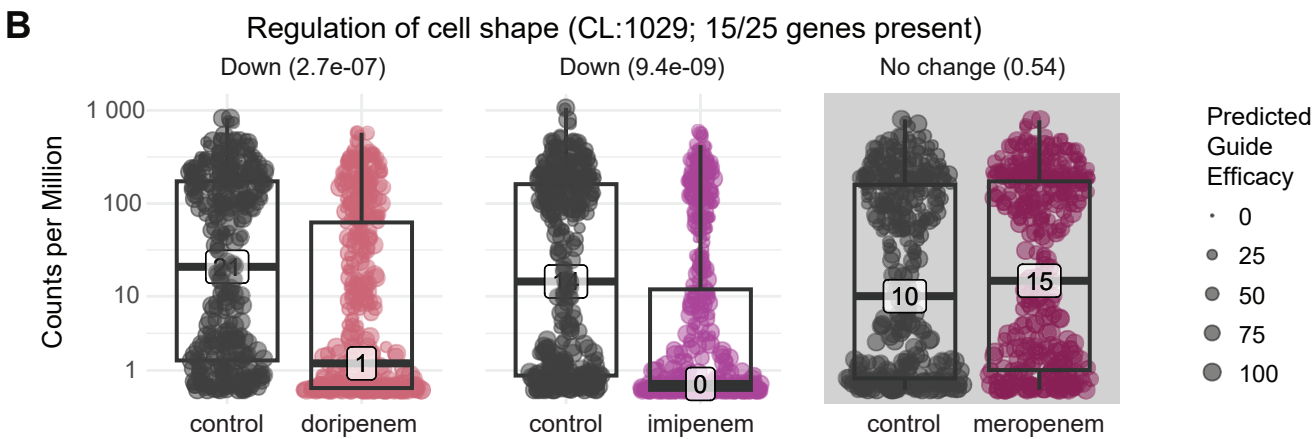

Supplement: S15 Fig — (A) Volcano plots of genes in meropenem, imipenem, or doripenem compared to no drug control. Genes with significant CG scores are shown in black. Meropenem has very few significant CG scores, suggesting insufficient chemical dosage. (B) Sina plots with boxplots of differential guide CPMs for target pathway in doripenem, imipenem, or meropenem. Chemical interactions compared to no chemical control (up, down, or no change) and FDRs are listed. Non-significant comparison for meropenem is shown with grey background. Doripenem and imipenem are significantly impacted. (PDF) [file pgen.1011642.s015.pdf]
