# Supplementary material for: Chemical genomics informs antibiotic and essential gene function in Acinetobacter baumannii
Source: PLoS Genet. 2025 Mar 28;21(3):e1011642. doi: 10.1371/journal.pgen.1011642 (PMC11975115; doi:10.1371/journal.pgen.1011642)
Supplement: S13 Fig — Sina plots with boxplots of differential guide CPMs for listed STRING identifiers in tetracycline-class antibiotics: (A) lipopolysaccharide (lipooligosaccharide in A. baumannii) transport or (B) ATP synthesis pathways. Guides are weighted by predicted efficacy, with perfect guides at 100. Chemical interactions compared to no chemical control (up, down, or no change) and FDRs are listed. Non-significant comparisons are shown with grey backgrounds. (C) Chemical structures of tetracycline, minocycline, and tigecycline. (PDF) [file pgen.1011642.s013.pdf]

S13

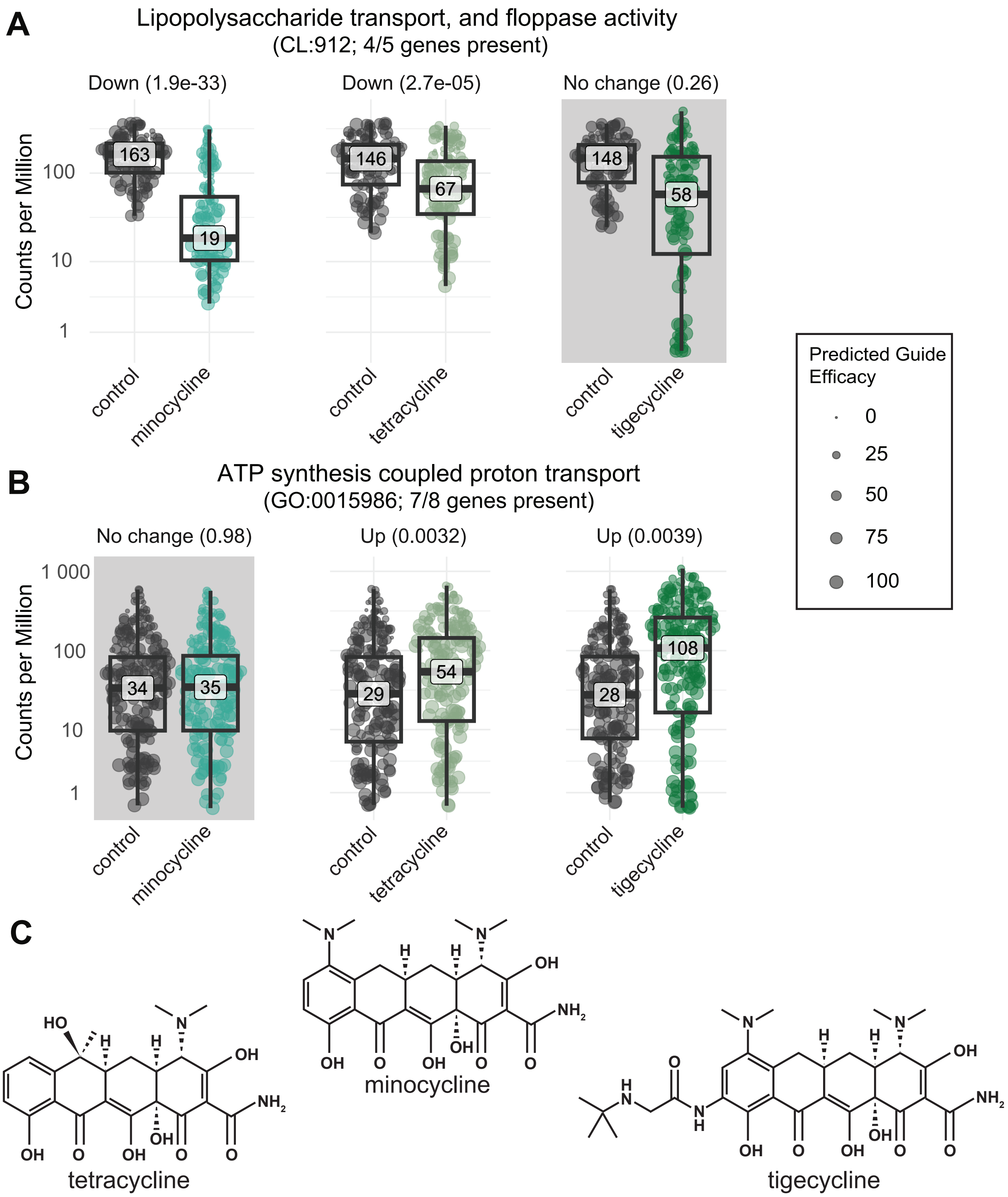
A
Lipopolysaccharide transport, and floppase activity
(CL:912; 4/5 genes present)
Down (1.9e-33)
Down (2.7e-05)
No change (0.26)
Counts per Million
100
10
1
163
19
146
67
148
58
control
minocycline
control
tetracycline
control
tigecycline
B
ATP synthesis coupled proton transport
(GO:0015986; 7/8 genes present)
No change (0.98)
Up (0.0032)
Up (0.0039)
1 000
100
10
1
34
35
29
54
28
108
Predicted Guide Efficacy
0
25
50
75
100
C
tetracycline
minocycline
tigecycline
